# When Adverse Childhood Experiences Present to a Statewide Child Psychiatry Access Program

**DOI:** 10.1007/s11414-023-09836-5

**Published:** 2023-03-31

**Authors:** Rebecca A. Ferro, Riley DiFatta, Kainat N. Khan, Kelly Coble, Shauna P. Reinblatt, Amie F. Bettencourt

**Affiliations:** 1 Johns Hopkins University School of Medicine, 550 N Broadway, 9Th Floor, Room 907, Baltimore, MD 21205 USA; 2 University of Maryland School of Medicine, 737 W Lombard Street, 4Th Floor, Room 424, Baltimore, MD 21201 USA

## Abstract

Many children experience adversity, yet few receive needed psychiatric services. Pediatric primary care providers (PCPs) are uniquely positioned to intervene but often lack training and resources to provide patients with adverse childhood experiences (ACEs) the psychiatric support they need. The current study examines characteristics of youth with and without ACEs who were the focus of PCP contacts with a statewide child psychiatry access program (CPAP). Compared to those without ACEs, patients with ACEs were more often receiving medication treatment at time of CPAP contact, prescribed two or more psychotropic medications, and diagnosed with two or more mental health disorders. Study findings indicate that patients with ACEs for whom PCPs sought CPAP support were experiencing more clinically severe and complex mental health concerns. These findings underscore the important role of CPAPs in supporting PCPs with pediatric patients who have ACEs and will inform training provided by CPAPs to PCPs.

## Introduction

Adverse childhood experiences (ACEs) are common, with approximately 43% of children having one or more.^1^ ACEs include childhood maltreatment, impaired caregiving,^2^ parental incarceration, and parental separation^3^—with more recent research supporting inclusion of community violence and economic hardship.^4^ ACEs are associated with long-term physical and mental health problems in adulthood.^5,6^ Outcomes of these early life adversities include increased risk for cancer, diabetes, heart disease, and mental illness and substance use.^3,7,8^ Early identification and intervention may help prevent these long-term outcomes;^9^ however, there is a significant gap between the need for and availability of effective interventions to support youth with ACEs.^10^

Pediatric primary care providers (PCPs) are often the first to see youth with ACEs and can play an important role in mitigating this risk. However, many pediatric PCPs are infrequently inquiring about ACEs and may not be familiar with the long-term implications.^11^ Additionally, pediatric PCPs face barriers to implementing routine ACE screening, including concerns of billing, limited time during appointments, lack of appropriate training in how to address ACEs if children screen positive, and the scarcity of mental health specialists.^12,13^

Due to the national shortage in child mental health specialists,^14^ timely referral from pediatric PCPs to specialized care is often not possible. Child psychiatry access programs (CPAPs) are one way to bridge this gap and support PCPs as they treat pediatric patients exposed to ACEs. CPAPs support PCPs in managing the mental health concerns of their pediatric patients through telephone consultation with child and adolescent psychiatrists, resource/referral networking, and continuing education, with some programs also offering direct-to-patient services (e.g., evaluation, brief psychotherapy) through co-located behavioral health or telehealth models.^15^ CPAPs can play a specific role in supporting pediatric PCPs in identifying and addressing ACEs, through training and consultations on screening and effective treatments as well as referrals for follow-up mental health care.^16^ Barclay and colleagues demonstrated that CPAPs receive a significant number of consultations about patients with trauma-related concerns, and this population presents with a high level of clinical severity.^17^ This previous research inspired the current study which expands to focus on adverse childhood experiences and analysis of consultation as well as co-located behavioral health service data.

The purpose of the current study is to understand the types and rates of ACEs reported by pediatric patients who were the focus of contacts with one statewide CPAP—Maryland Behavioral Health Integration in Pediatric Primary Care (BHIPP). This study explores the characteristics and service use of these youth with and without ACEs, including (1) demographics, clinical severity, and presenting concerns, (2) rates and types of treatment received prior to BHIPP contact, and (3) types of psychiatric medications employed. In light of the fact that the US Department of Health and Human Services has expanded funding to CPAPs nationwide,^18^ these findings are even more critical in guiding future CPAP training and service offerings and in identifying treatment gaps for ACE exposed youth.

## Methods

BHIPP provides an array of services including consultation and co-located social work services. BHIPP’s warm line is staffed by licensed master's-level behavioral health consultants, from whom PCPs can request general information, resource/referral networking tailored to patients, or consultation with a child and adolescent psychiatrist. BHIPP also provides embedded social work services in selected primary care practices where master’s level social work interns conduct screening and brief behavioral health interventions (i.e., up to 6 sessions). Pediatric patients are connected with this service through PCP or self/family referral. This study is a secondary analysis of programmatic data collected during the provision of normal BHIPP services.

From October 2012 through September 2020, 8228 patient-specific BHIPP contacts were completed through telephone consultation with a BHIPP child and adolescent psychiatrist and in-person visits with an embedded BHIPP social work intern. These contacts pertained to pediatric patients seen in a primary care setting in the state of Maryland. As of September 2020, there were 1127 pediatric providers across the state who had engaged with BHIPP through enrolling for services, calling the warm line, or both. Co-located social work services were provided in selected primary care practices in one rural region of Maryland by an average of eight BHIPP interns per academic year. Data on patients above 24 years old (*N* = 24) or repeat contacts concerning the same patient (*N* = 3211 due to multi-visit nature of social work co-location) were excluded from analyses. This study includes the remaining 4993 unique patient-specific BHIPP contacts for psychiatric consultation (1970 calls) and co-located social work services (3023 initial visits). IRB approval was obtained from Johns Hopkins University, University of Maryland, and Maryland’s Department of Health.

During all patient-specific contacts, BHIPP staff collected de-identified data on patient demographics (e.g., gender, race/ethnicity), insurance type (e.g., private, public), presenting concerns (e.g., anxiety, depressed mood), and prior history of behavioral health treatment. Providers or families (for co-located social work visits) were asked if the patient had experienced any adverse childhood experiences or trauma. BHIPP staff selected the reported ACE(s) or trauma from a list of 23 options such as child maltreatment, separation from caregiver, and loss of a loved one (see Table 1 for full list). After services were rendered, BHIPP staff recorded the following: diagnostic impression(s) of the patient, a rating of the patient’s clinical severity on a 7-point scale from normal to extremely ill using the Clinical Global Impression Score (CGI-S),^19^ and treatment recommendations. Patients with a CGI-S above 4 were considered clinically severe. Patients with more than one mental health diagnosis were considered to have comorbid diagnoses. Study data were collected and managed using Research Electronic Data Capture (REDCap) tools hosted at Johns Hopkins University.^20,21^

**Table 1:** Adverse childhood experiences disclosed in pediatric primary care by patients who were the subject of BHIPP contacts

Adverse experience/trauma	Overall		Co-location		Consultation	
	*N*	%	*N*	%	*N*	%
Separation from primary caregiver	212	4.2	99	3.3	113	5.7
Loss of loved one	150	3.0	67	2.2	83	4.2
Impaired caregiver	94	1.9	36	1.2	58	2.9
Arrest/incarceration of parent/caregiver	72	1.4	44	1.5	28	1.4
Bullied	70	1.4	42	1.4	28	1.4
Witnessed domestic violence	70	1.4	31	1.0	39	2.0
Sexual abuse	66	1.3	28	0.9	38	1.9
Physical abuse	54	1.1	18	0.6	36	1.8
Neglect	48	1.0	25	0.8	23	1.2
Emotional abuse	43	0.9	18	0.6	25	1.3
Out of home placement	39	0.8	13	0.4	26	1.3
Hospitalization	30	0.6	7	0.2	23	1.2
Accident	25	0.5	13	0.4	13	0.7
Invasive medical procedures	20	0.4	3	0.1	17	0.9
Divorce	18	0.4	5	0.2	13	0.7
Homelessness	16	0.3	2	0.1	14	0.7
Assaulted	13	0.3	2	0.1	11	0.6
Witnessed community violence	6	0.1	-	-	6	0.3
House fire	4	0.08	3	0.1	1	0.1
Attacked by animal	3	0.06	1	0.03	2	0.1
Accidental burning	2	0.04	-	-	2	0.1
Kidnapping	2	0.04	2	0.1	-	-
Natural disaster	2	0.04	-	-	2	0.1
Family denies trauma history*	431	8.6	–-	–-	431	21.9
Unknown to provider	880	17.6	4	0.1	876	44.5
Total sample	4993	100	3,023	100	1970	100

percentages are based on total sample (*N* = 4113). Patients may endorse more than one adverse experience or trauma. “-” represents no patient reports of a type of ACE. *N* = 125 patients were diagnosed with a trauma and stressor related disorder but no information on a history of trauma or ACEs was available. Cases where adverse experience history was unknown to provider were not included in study analyses

^*^ “Family denies trauma history” was an option for consultation data only

### Statistical analyses

Descriptive statistics and chi-square analyses were used to examine patient characteristics, differences between patients with and without ACEs, and differences among patients with ACEs who were the subject of BHIPP consultation versus co-located social work services.

### Missing data

A subset of patient-specific contacts (17.6%, *N* = 880; Table 1) had an unknown history of ACEs and were excluded from subsequent analyses. Those excluded differed from the patient-specific contacts included in this study on demographic and clinical characteristics. Specifically, chi-square tests revealed that those patients with unknown ACE history were more often male *χ*^2^(6) = 99.89, *p* < 0.001 and between the ages of 13 and 18 *χ*^2^(8) = 202.11, *p* < 0.001, than those with information on ACE history. In terms of clinical characteristics, those with unknown ACE history more often reported attention/concentration problems *χ*^2^(2) = 26.00, *p* < 0.001; were more often diagnosed with attention deficit/hyperactivity disorder (ADHD) *χ*^2^(2) = 156.03, *p* < 0.001; had more comorbid mental health diagnoses *χ*^2^(2) = 414.72, *p* < 0.001; and were more often rated as clinically severe (27.8% CGI-S > 4) *χ*^2^(4) = 264.21, *p* < 0.001. Furthermore, those with unknown ACE history were more often receiving medication treatment *χ*^2^(2) = 702.46, *p* < 0.001 and outpatient psychotherapy *χ*^2^(2) = 187.58, *p* < 0.001; had higher rates of polypharmacy *χ*^2^(12) = 507.30, *p* < 0.001; and were more often recommended by BHIPP staff for a medication evaluation/change *χ*^2^(2) = 1209.31, *p* < 0.001 than those included in the study. Thus, the final analytic sample includes 4113 patient-specific contacts with information on ACEs.

## Results

Among the 4113 unique patient-specific contacts included in this study, 19.6% (*N* = 807) focused on children with a history of ACEs or a diagnosis of a trauma and stressor-related disorder (i.e., acute stress disorder or PTSD), hereafter referred to as the “ACE group.” The remaining patients (*N* = 3306) who did not report a history of ACEs or trauma are referred to as the “no ACE group” (Table 1). Among those reporting ACEs, the most frequent were separation from primary caregiver (*N* = 212; 31.1%), loss of a loved one (*N* = 150; 22.0%), and impaired caregiver (*N* = 94; 13.8%). Among those reporting ACEs (*N* = 682), 67.2% reported one ACE, 20.1% reported two ACEs, and 12.8% reported three or more ACEs.

Overall, the most frequently endorsed presenting problems for those in the ACE group were anxiety (39.4%), behavior problems at home (30.1%), and depressed mood (26.8%), see Table 2. Significant differences in presenting problems were found in the ACE group by patient age. Compared to other age groups, patients below age 6 most frequently presented with behavior problems at home (55.5%) *χ*^2^(3) = 96.38, *p* < 0.001 and aggression (32.8%) *χ*^2^(3) = 54.22, *p* < 0.001. For patients aged 6–12, behavior problems at school (27.5%) *χ*^2^(3) = 40.87, *p* < 0.001 and attention/concentration problems (22.2%) *χ*^2^(3) = 27.28, *p* < 0.001 were the most frequently reported. Patients between 13 and 18 years old more frequently presented with depressed mood (52.7%) *χ*^2^(3) = 172.83, *p* < 0.001 and suicidal thoughts or gestures (16.5%) *χ*^2^(3) = 38.91, *p* < 0.001, compared to those in other age groups. Finally, young adults (age 19 and up) more frequently presented with anxiety (70.6%) *χ*^2^(3) = 64.79, *p* < 0.001, compared to younger age groups.

**Table 2:** Presenting problems and diagnosis for pediatric patients with and without ACEs who were the subject of BHIPP contacts

	ACE group		No ACE group		Chi-square value
	N	%	N	%	
Presenting problem					
Anxiety	318	39.4	1,083	32.8	12.76***
Behavior problems at home	243	30.1	819	24.8	9.65**
Depressed mood	216	26.8	537	16.2	48.02***
Behavior problems at school	163	20.2	575	17.4	3.47
Aggression	140	17.3	341	10.3	31.07***
Attention/concentration	126	15.6	606	18.3	3.27
Sleep problems	103	12.8	201	6.1	42.33***
Impulsive behaviors	89	11.0	161	4.9	43.10 ***
Hyperactivity	74	9.2	273	8.3	.670
Adjustment	74	9.2	230	7.0	4.64*
Parent–child conflict	71	8.8	175	5.3	14.17***
Underachievement at school	63	7.8	153	4.6	13.17***
Worries/fears	59	7.3	190	5.7	2.79
Diagnosis					
Anxiety disorder	328	40.6	1,133	34.3	11.50***
Major depressive disorder	167	20.7	356	10.8	57.58***
Dysthymia	9	1.1	29	0.9	.40
Mood disorder	55	6.8	140	4.2	9.57**
Bipolar disorder	17	2.1	19	0.6	17.54***
ADHD	244	30.2	880	26.6	4.27*
Autism	34	4.2	138	4.2	.002
Disruptive disorder/ODD	106	13.1	237	7.2	30.21***
Adjustment disorder	58	7.2	159	4.8	7.34**
Substance use disorder	8	1.0	18	0.5	2.06
Psychotic disorder	10	1.2	11	0.3	10.49***
Eating disorder	3	0.4	33	1.0	2.93
Learning disorder	23	2.9	87	2.6	.12
Developmental disorder	31	3.8	37	1.1	29.56**
Comorbid medical	14	1.7	33	1.0	3.12

Only presenting concerns endorsed by 5% or more of the total sample are included in the table; additional categories were avoidance, compulsive behavior, cutting/self-injury, delusions, destructive behavior, developmental delay/concerns, dissociation, eating/feeding problems, elimination problems, emotional dysregulation, expansive mood, grief, hallucinations, homicidal thoughts/gesture, hurting animals, labile mood, learning disability/difficulties, legal problems, obsessive thoughts, reckless/risky behavior, relationship issues, sexual acting out, sexual/gender identity, somatic complaints, substance use, suicidal thoughts/gestures, suicide attempt, tics (motor/vocal), and truancy

^***^p ≤ .001, **p ≤ .01, *p ≤ .05

### Comparison of patients with and without ACE exposure for whom PCPs sought BHIPP services

Patients in the ACE group who were the subject of BHIPP contacts were more often female (54.5%), ages 6–12 (44.1%), and Caucasian (55.5%) as compared to those in the no ACE group (Table 3). Those in the ACE group were more often publicly insured (48.2%) compared to those without ACEs (35.1%), *χ*^2^(2) = 65.57, *p* < 0.001. Numerous significant differences in the presenting problems were identified between those in the ACE and no ACE groups, for which the ACE group more frequently endorsed problems (Table 2). Patients in the ACE group more often presented with aggression (17.3%), *χ*^2^(1) = 31.07, *p* < 0.001; sleep problems (12.8%), *χ*^2^(1) = 42.33, *p* < 0.001; and impulsivity (11.0%), *χ*^2^(1) = 43.10, *p* < 0.001. There were also significant differences in diagnostic impressions across the ACE and no ACE groups (Table 2). Among the ACE group, anxiety disorders were the most common diagnostic impression (40.6%), followed by ADHD (30.2%) and major depression (20.7%); while these same diagnostic impressions were most common among the no ACE group, the rates were lower for each disorder (34.3%, 26.6%, 10.8%, respectively).

**Table 3:** Characteristics of pediatric patients with and without ACEs who were the subject of BHIPP contacts

Demographics	ACE group		No ACE group		Chi-square value
	*N*	%	*N*	%	
Gender					42.52***
Male	357	44.2	1509	45.6	
Female	440	54.5	1570	47.5	
Other	-	-	2	0.1	
Missing	10	1.2	225	6.8	
Age					41.99***
0 to 5	137	17.0	613	18.5	
6 to 12	356	44.1	1322	40.0	
13 to 18	273	33.8	1057	32.0	
19 and up	34	4.2	108	3.3	
Missing	7	0.9	206	6.2	
Race/ethnicity					38.23***
Caucasian	448	55.5	1,943	58.8	
African American	161	20.0	513	15.5	
Asian	11	1.4	21	0.6	
American Indian	-	-	3	0.1	
Native Hawaiian	-	-	3	0.1	
Latino	58	7.2	194	5.9	
Biracial	28	3.5	92	2.8	
Other	19	2.4	34	1.0	
Missing	82	10.2	503	15.2	
Insurance type					65.57***
Public only	389	48.2	1161	35.1	
Private or both private and public	246	30.5	999	30.2	
None/unknown	172	21.3	1146	34.7	
Total	807	100	3306	100	

^***^p ≤ .001

Those in the ACE group were rated more clinically severe by BHIPP staff *χ*^2^(2) = 142.54, *p* < 0.001, than those in the no ACE group, with 24.4% of patients in the ACE group receiving a CGI-S > 4. Patients in the ACE group were more often identified as having comorbid diagnoses (diagnosed with two or more mental health disorders), (50.1%) *χ*^2^(1) = 229.83, *p* < 0.001, and were more often already receiving behavioral health treatment at the time of BHIPP contact, (48.7%) *χ*^2^(2) = 51.61,* p* < 0.001, than those in the no ACE group. Among those already receiving treatment in the ACE group, the most frequent treatments were medication (27.0%) and outpatient psychotherapy (20.2%; Table 4). Compared to those in the no ACE group, patients in the ACE group were more often receiving medication at the time of BHIPP contact (27.0%) and were more likely to be prescribed multiple medications, (12.4%) *χ*^2^(3) = 68.92, *p* < 0.001, with the most frequent medications prescribed being stimulants (15.1%) and SSRIs (13.4%). BHIPP recommendations made at the time of contact also varied by group with those in the ACE group more often recommended for referral to mental health or community resources (49.8%), *χ*^2^(1) = 141.96, *p* < 0.001 and medication management (36.1%), *χ*^2^(1) = 143.94, *p* < 0.001, whereas in-office behavioral interventions were more commonly recommended for those in the no ACE group (40.7%) *χ*^2^(1) = 20.39, *p* < 0.001.

**Table 4:** Behavioral health treatments received by patients with and without ACEs at time of BHIPP service contact

Current treatment	ACE group		No ACE group		Chi-square value
	*N*	%	*N*	%	
Medication treatment	218	27.0	487	14.7	68.90***
In-office behavioral intervention	47	5.8	416	12.6	29.67***
Outpatient psychotherapy	163	20.2	271	8.2	98.98***
School-based services	70	8.7	173	5.2	13.82***
Family education and support	17	2.1	69	2.1	.001
Early childhood mental health services	5	0.6	25	0.8	.17
Ancillary services	7	0.9	10	0.3	5.03*
Child find or special Ed services	5	0.6	11	0.3	1.38
Case management/family navigation	2	0.2	10	0.3	.07
Mental health consultation	1	0.1	9	0.3	.59
Psychological evaluation	2	0.2	7	0.2	.04
In-home services	3	0.4	3	0.1	3.52
Intensive outpatient	1	0.1	3	0.1	.07
Emergency room/crisis response	4	0.5	5	0.2	3.52
Inpatient or residential treatment	2	0.2	4	0.1	.72

Early childhood mental health services include early learning centers, home visiting, early childhood mental health clinic, and infants and toddlers/Part C

^***^p ≤ .001, *p ≤ .05

### Comparison of characteristics of the ACE group by BHIPP service received

Characteristics of patients in the ACE group were also examined across BHIPP service types to identify any differences in patient characteristics between those who were the subject of telephone consultation with a child and adolescent psychiatrist and co-located social work services. Those with ACEs receiving co-located social work services were more often female (59.6%) and under the age of 6 (22.1%) as compared to those who were the subject of consultation. However, the majority of patients across both services were 6–12 years old (co-located social work visits: 48.1%; consultation: 40.4%). Numerous significant differences were found between the frequencies of ACEs reported across service types. Of those receiving co-located social work services, 9.4% reported at least one ACE, whereas 36.4% of those receiving services through consultation reported at least one ACE *χ*^2^(3) = 425.30, *p* < 0.001. In nearly all cases, a higher proportion of patients who were the subject of consultation services reported each type of ACE including having an impaired caregiver *χ*^2^(1) = 4.18, *p* = 0.041; experiencing physical abuse *χ*^2^(1) = 5.13, *p* = 0.024; and hospitalization *χ*^2^(1) = 6.97, *p* = 0.008, relative to co-located social work services (Table 1). Patients who were the focus of co-located social work services more often reported arrest/incarceration of caregiver *χ*^2^(1) = 5.28, *p* = 0.022 and being bullied *χ*^2^(1) = 4.27, *p* = 0.039. Those with ACEs receiving services through consultation were also rated as more clinically severe (37.3% CGI-S > 4) *χ*^2^(1) = 78.32, *p* < 0.001 and identified as having more comorbid diagnoses (33.5% with 3 or more diagnoses) *χ*^2^(1) 57.86, *p* < 0.001 (Table 5). At the time of BHIPP contact, those who were the subject of consultation were more often receiving behavioral health treatment, *χ*^2^(2) = 518.90, *p* < 0.001, and more often prescribed multiple psychiatric medications, compared to those receiving co-located social work services.

**Table 5:** Characteristics of patients with ACEs who were the subject of BHIPP contacts by BHIPP service type

	Co-location		Consultation		Chi-square value
	*N*	%	*N*	%	
Severity					
1–2	133	34.2	2	0.5	164.38***
3–4	214	55.0	237	56.7	.23
5–7	41	10.5	156	37.3	78.32***
Missing	1	0.3	23	5.5	
Comorbidity					
No diagnosis	108	27.8	23	5.5	73.43***
1 diagnosis	147	37.8	125	29.9	5.61*
2 diagnoses	91	23.4	130	31.1	6.02*
3 or more diagnoses	43	1.3	140	33.5	57.86***
Medication type					
Antidepressants	-	-	10	2.4	9.42**
Antipsychotic	1	0.3	26	6.2	22.16***
Stimulant	22	5.7	100	23.9	52.40***
Mood stabilizer	1	0.3	21	5.0	17.27***
Non-stimulant ADHD	11	2.8	64	15.3	37.25***
Sedative	1	0.3	16	3.8	12.46***
SSRI	19	4.9	89	21.3	46.79***
Other	10	2.6	25	6.0	5.65*
Polypharmacy					
No medication	335	86.1	198	47.4	143.45***
1	45	11.6	129	30.9	
2	8	2.1	58	13.9	
3 or more	1	0.3	33	7.9	

the severity ratings for the CGI-S are described as follows: 1 = normal, not at all ill, 2 = borderline mentally ill, 3 = mildly ill, 4 = moderately ill, 5 = markedly ill, 6 = severely ill, 7 = among the most extremely ill patients

^***^p ≤ .001, **p ≤ .01, *p ≤ .05

## Discussion

The study findings indicate that pediatric patients who are the subject of CPAP contact and report a history of ACEs present to primary care offices with higher clinical severity and complexity than those who do not have a history of ACEs. Specifically, the patients with ACEs who are the focus of CPAP contacts have higher rates of mental health diagnoses, are more frequently diagnosed with multiple comorbid mental health conditions, are more often prescribed psychiatric medications, and are more often taking multiple psychiatric medications. Nearly 20% of the current sample reported at least one ACE with the most common being separation from primary caregiver, loss of a loved one, and impaired caregiver. In addition, relative to co-located social work services, patients who were the focus of consultations reported nearly four times the number of ACEs and demonstrated greater clinical severity and complexity of mental health concerns.

However, research has shown that routine screening for ACEs in pediatric primary care is not a common practice.^11^ Lack of routine screening is likely one of the primary reasons for which the current study had a portion of patients with missing data on ACEs. We posit two potential explanations for the differences between those included in this study and this missing data—for which even higher clinical severity was found. First, it is possible that these patients did not disclose a history of ACEs to PCPs as they were already receiving behavioral health treatments and may not have felt that the disclosure of ACE history was necessary. Second, the most frequent diagnosis among the unknown group was ADHD. While many of these cases are likely to have an accurate ADHD diagnosis, it is also possible that some of these patients are presenting with posttraumatic stress symptoms that may be misdiagnosed as ADHD (decreased attention/concentration, difficulty with emotion regulation, and hyperarousal). Whereas some research suggests that trauma is a risk factor for ADHD, others have found that trauma is frequently misdiagnosed as ADHD,^22^ thus underscoring the importance of thorough screening for ACEs and a more in-depth assessment of mental health concerns to support appropriate diagnosis and treatment.

There are some limitations related to the method for collecting data on ACE exposure. Providers and patients/families were not necessarily presented with a list of ACEs but were rather asked a general question about ACE exposure. The knowledge of what experiences constitute ACEs may vary by the provider, and patients/families may feel uncomfortable disclosing this information. Likewise, pediatric PCPs may feel uncomfortable inquiring about ACEs or may not have time to address ACE-related concerns. Additionally, data from consultations are PCP-reported and not directly collected from the patient/family—creating a filter in which information may be lost. It seems likely that the current sample of pediatric patients had a higher number of ACEs than were actually reported to PCPs or BHIPP staff, as the rate of ACEs in the current study is much lower than other community samples.^1,23^ For example, in 2018–2019, the rate of ACEs among children in Maryland was 38.4%, considerably higher than the 19.6% reported in the current sample.^23^ The majority of patients in the current study only reported experiencing one ACE which may be accurate or may be another sign of underreporting. Regardless, prior work suggests that experiencing even one ACE is enough to contribute to negative mental and physical health outcomes, though a dose–response relationship is observed in that those with multiple ACEs are at an increased risk for more negative outcomes.^3,24^ It is also important to note that ACEs have differential impacts. Recent research suggests that household dysfunction is more highly associated with health risks, while child maltreatment is more highly associated with psychological difficulties.^25^ In addition, the pediatric patients included in this study have been identified as having some level of mental health concerns necessitating a BHIPP consultation call or referral to BHIPP co-located social work services—making this sample not generalizable to the population of youth presenting to primary care.

Future research should address best practices for inquiring about ACEs in pediatric primary care including how to help families feel comfortable disclosing ACEs and strategies for helping pediatric PCPs incorporate ACE screening into routine visits. In a recent study by Thakur and colleagues, surveying patients about ACEs in aggregate (i.e., “indicate the number of experiences that have occurred”) instead of by individual ACE resulted in more ACEs being reported.^26^ Future work should examine methods for ACE screening across settings and with diverse patient populations. Results from the current study show that Caucasian female pediatric patients most often reported ACEs; however, research has demonstrated a disproportionate trend in ACE exposure for systematically marginalized populations with Black and Latinx youth experiencing more cumulative ACEs than their White peers.^27^ Cultural implications for disclosure of ACEs in the pediatric primary care setting should be studied to inform culturally responsive care, to reduce ethnic and racial disparities in ACE exposure, and increase access to effective treatments. One potential way to increase culturally responsive care may be to employ diverse, bilingual support staff that may assist with screening and referral services.^28^ Future work should explore the impacts of such practices on disclosure rates of ACEs within the pediatric primary care setting. Finally, future research should explore the impact of receipt of CPAP services on changes to the care PCPs provide to their patients who have a history of ACEs.

## Implications for Behavioral Health

The current study’s findings will help inform training provided by CPAPs to pediatric PCPs across the country. Specifically, findings indicate that additional training is needed to increase pediatric PCP knowledge of the types of ACEs, which are most common, and the psychosocial, emotional, and physical consequences of ACE exposure for child health and development. Training could increase provider comfort with asking about ACEs by supporting pediatric PCPs in identifying what screening tools to use, how to respond to patients’ report of ACEs, and best practices for follow-up. Providers also need support in the management of mental health concerns related to ACE exposure and identification of appropriate community resources—child psychiatry access programs like BHIPP reflect one model for providing this support.^15^ These findings about Maryland’s CPAP are relevant to nationwide efforts by CPAPs to bridge the gap between the need for and access to mental health treatment for ACE-exposed youth.

Mental health promotion for pediatric patients with a history of ACEs is in critical need.^10^ Routine screening is imperative to identify youth at risk of developing significant mental and physical health concerns. As of January 2020, California launched universal ACE screening in the context of primary care, and providers are now able to receive reimbursement for using the Pediatric ACEs and Related Life Events Screener.^29^ In order to successfully implement routine ACE screening, pediatric providers nationwide will need access to reimbursement and support in appropriately responding to and providing care for youth with ACE exposure. CPAPs can provide training and technical assistance to pediatric PCPs in screening for ACEs to help promote PCPs’ use of validated ACE screening tools with their patients to inform the consultation process and ensure that appropriate treatment options are provided.

Avenues for supporting youth with ACE exposure in the context of primary care include continuous monitoring of youth behavioral and physical health and referral to specialty mental health care (including cognitive behavioral therapy and parenting skills training) and community supports (e.g., food banks, financial resources, mental health care).^30^ Pediatric PCPs can also be trained to provide brief in-office interventions that include psychoeducation on how ACEs impact health and anticipatory guidance for caregivers regarding developmental periods that are typically challenging.^31^ CPAPs could provide such training and support to pediatric PCPs in implementing these interventions and identifying available mental health and community resources. Finally, collaborative care practices between behavioral health providers and pediatric PCPs regarding treatment planning can improve the continuity of care for youth exposed to ACEs.
